# Comparative Analysis of Conformational Dynamics and Nucleotide Binding in miniGαi1 and Full-Length Gαi1

**DOI:** 10.3390/ijms27219819

**Published:** 2026-11-01

**Authors:** Longhan Duan, Ka Young Chung

**Affiliations:** School of Pharmacy, Sungkyunkwan University, 2066 Seoburo, Jangan-gu, Suwon 16419, Republic of Korea

**Keywords:** G protein, miniG, conformation, GTP

## Abstract

Mini G proteins (miniG proteins) are engineered Gα constructs lacking the α-helical domain (AHD) and are widely used to stabilize active G protein-coupled receptor (GPCR) conformations for structural and biochemical studies. However, the intrinsic conformational and nucleotide-binding properties of miniG proteins remain incompletely understood relative to their full-length counterparts. Here, we compared miniGαi1 and full-length Gαi1 using fluorescence-based nucleotide-binding assays and hydrogen–deuterium exchange mass spectrometry (HDX-MS). BODIPY–FL–GTP assays showed that miniGαi1 interacted with GTP more rapidly and produced higher fluorescence intensity than full-length Gαi1. Unlike full-length Gαi1, miniGαi1 did not exhibit fluorescence decay following nucleotide binding, suggesting impaired or absent GTP hydrolysis activity. HDX-MS analysis demonstrated globally higher deuterium uptake in miniGαi1, particularly in regions associated with nucleotide binding and GPCR interaction. These results indicate that miniGαi1 adopts a more solvent-accessible and conformationally dynamic state than GDP-bound full-length Gαi1. Together, our findings provide biochemical and structural insight into the intrinsic properties of miniG proteins.

## 1. Introduction

**Figure 1:**
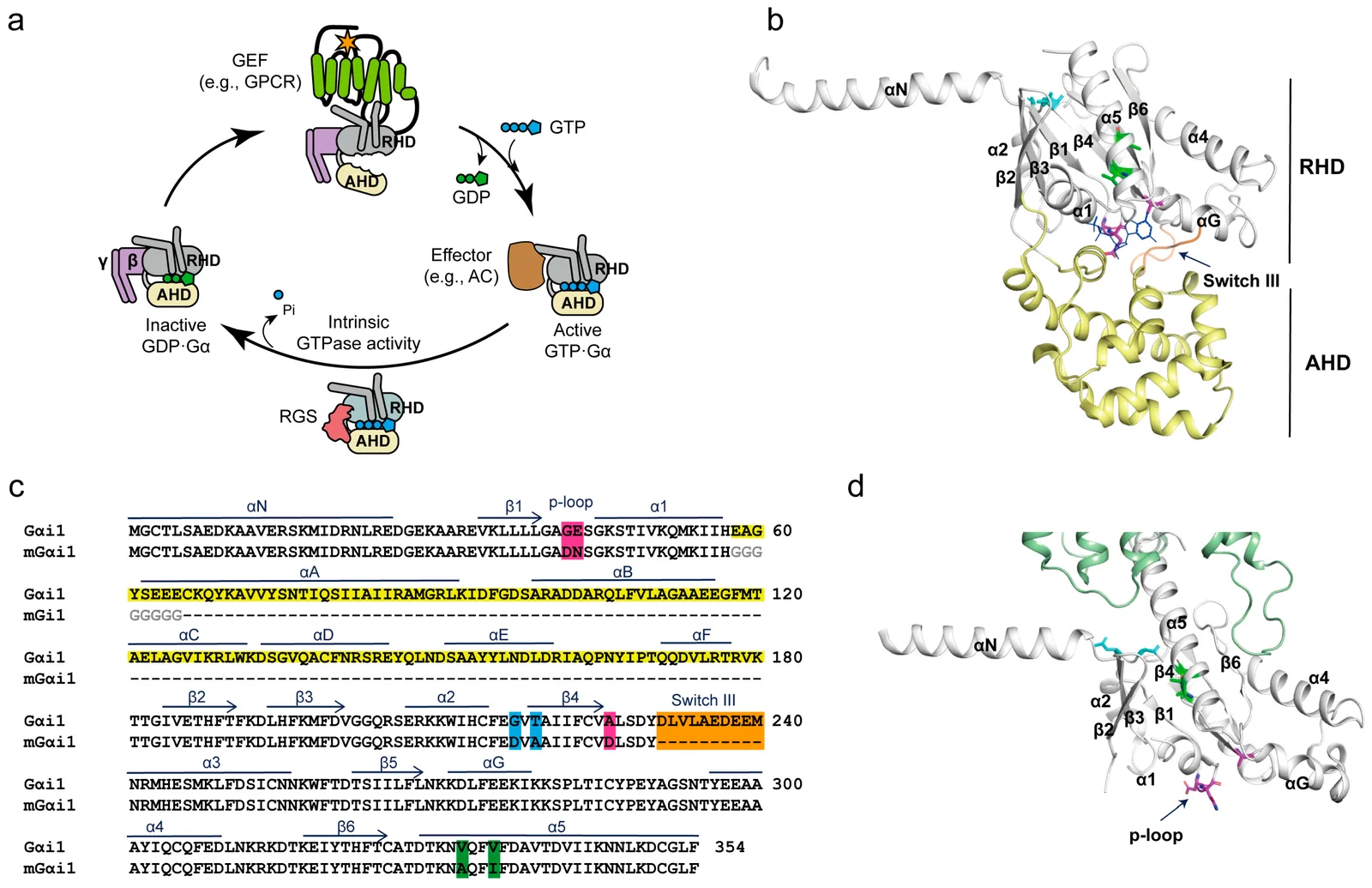
miniGαi1 construct generation. (**a**) Schematic illustration of the G protein activation cycle. Agonist (orange star)-bound activated GPCRs function as guanine nucleotide exchange factors (GEFs), promoting GDP release from inactive GDP-bound heterotrimeric G proteins and facilitating GTP binding. GTP-bound Gα subsequently interacts with downstream effectors, such as adenylyl cyclase (AC), to initiate intracellular signaling. Owing to its intrinsic GTPase activity, GTP-bound Gα hydrolyzes GTP to GDP, thereby terminating signaling. This process is accelerated by regulator of G protein signaling (RGS) proteins. During G protein activation, the α-helical domain (AHD) undergoes a conformational rearrangement relative to Ras-homology domain (RHD). (**b**,**d**) Structures of full-length Gαi1 and miniGαi1 derived from the crystal structure of Gαi1 (PDB: 1gp2) and the cryo-EM structure of the ADGRF1–miniGαi1 complex (PDB: 7wu4), respectively. The RHD is colored in gray, and the AHD in pale yellow. Engineered mutation sites in miniGαi1 are highlighted as colored sticks, and the switch III deletion is indicated by an orange cartoon. GDP is shown as blue lines. (**c**) Sequence alignment of full-length Gαi1 and miniGαi1. The seven engineered mutations and switch III deletion are highlighted in different colors, and the deleted AHD region is highlighted in yellow. The linker between α1 and β2 in miniGαi1 is shown in grey.

The heterotrimeric G proteins are composed of three distinct subunits, α, β, and γ. Binding of guanosine diphosphate (GDP) to Gα stabilizes the Gαβγ heterotrimer in its basal state. When activated G protein-coupled receptors (GPCRs) interact with Gα, it promotes GDP release, followed by guanosine triphosphate (GTP). GTP binding induces conformational changes in Gα, leading to its dissociation from the receptor and Gβγ. The GTP-bound Gα subsequently interacts with effector proteins to initiate downstream signaling pathways. Intrinsic GTPase activity of Gα hydrolyzes GTP into GDP, thereby promoting re-association of Gα with Gβγ and restoration of the basal state (Figure 1a) [1].

Heterotrimeric G proteins are classified into four subfamilies (Gs, Gi/o, Gq/11, and G12/13) based on sequence similarity and downstream signaling properties of Gα [2]. Structurally, Gα consists of two domains: a Ras-homology GTPase domain (RHD) and an α-helical domain (AHD). GDP or GTP is positioned at the interface between these two domains mainly interacting at RHD (Figure 1a,b) [3]. Activated GPCRs primarily interact with the RHD and induce conformational rearrangements that facilitate GDP release. One of the major structural changes during this process is the displacement of the AHD, resulting in an open conformation of Gα (Figure 1a). In the nucleotide-free state, the AHD remains highly mobile and displaced from the RHD, whereas GTP binding promotes re-closure of the two domains.

Notably, the AHD is unique to Gα and is absent in small GTPases. Small G proteins are fully functional without the AHD, and the RHD alone retains many canonical functions of G proteins, including GTP hydrolysis and interactions with receptors, Gβγ subunits, and downstream effectors. Based on these observations, engineered Gα constructs lacking the AHD, termed miniG proteins (miniGα), were developed (Figure 1c,d) [4,5,6].

The first miniG protein, miniGαs, was developed through sequence comparison with Arl2, a member of the small GTPase family, to identify structural elements that could be modified to improve the stability of the receptor-bound conformation [4]. To preserve GPCR-coupling capability while increasing protein stability and uncoupling GPCR interaction from GDP/GTP exchange, several additional mutations were introduced along with deletion of the AHD [4,5]. Mutations in the α5 helix (Figure 1b,d, green sticks) were introduced to stabilize the receptor-bound state and uncouple GPCR binding from nucleotide release. Additional mutations in the nucleotide-binding pocket (Figure 1b,d, magenta sticks), together with deletion of the switch III region (Figure 1b, orange cartoon), further enhanced the structural stability of miniG proteins. These mutations were conserved across other miniG proteins, whereas miniGαq was developed as a miniGαs/q chimera because of the poor expression of miniGαq in *E. coli* [5].

These engineering strategies have enabled miniG proteins to serve as useful tools for investigating active GPCR conformations and signaling. Owing to their enhanced stability and ability to stabilize active GPCR conformations, miniG proteins have been widely used in structural and biochemical studies. In particular, they have facilitated high-resolution structural analyses of GPCR–G protein complexes that are otherwise difficult to obtain using full-length G proteins, in part because of the conformational flexibility of the AHD [7,8,9]. More recently, fluorescently labeled miniG proteins have been used to probe GPCR activation in live cells by monitoring changes in their cellular localization following receptor stimulation [6].

Despite their extensive use in the GPCR field, the intrinsic biochemical and conformational properties of miniG proteins remain incompletely characterized in comparison with their full-length counterparts. In particular, the contributions of AHD deletion and the additional engineering modifications including stabilizing mutations in the α5 helix and nucleotide-binding pocket, as well as switch III deletion, to the biochemical properties and conformational dynamics of miniG proteins have not been systematically examined.

In this study, we sought to characterize the nucleotide interaction properties and conformational dynamics of miniGαi1 relative to full-length Gαi1 using complementary nucleotide assays and hydrogen–deuterium exchange mass spectrometry (HDX-MS). We focused on Gαi1 because it is a widely expressed member of the Gi family, which comprises several subtypes, including Gαi1, Gαi2, Gαi3, GαoA, Gαz, and Gαt, with distinct tissue distribution and receptor coupling profiles. Moreover, Gi-family proteins account for approximately 71% of reported GPCR–G protein coupling pairs catalogued in GPCRdb, underscoring the importance of understanding the molecular basis of GPCR–Gi coupling. By comparing miniGαi1 with its full-length counterpart, we aimed to determine how AHD deletion and additional engineering modifications collectively influence nucleotide interactions and conformational dynamics, with potential implications for miniG constructs derived from other G protein families.

## 2. Results

### 2.1. Construction of miniGαi1

In this study, we adopted a previously reported miniGαi1 construct containing mutations in the nucleotide-binding pocket (G42N, E43N, and A226D), the α5 helix (V332A and V335I), and α2/β4 (G217D and T219A), along with truncation of the switch III region (from D231 to M240) (Figure 1c). The positions of these mutations are highlighted as sticks and cartoons in the structures of GDP-bound full-length Gαi1 (Figure 1b) and miniGαi1 bound to Adhesion G Protein-Coupled Receptor F1 (ADGRF1) (Figure 1d).

To date, high-resolution structures of miniGα are available only in complexes with GPCRs. For example, the miniGαi1 structure shown in Figure 1d (white) was solved in complex with the receptor ADGRD1 (green cartoon). Consequently, the intrinsic conformation of miniGαi1 in the absence of a GPCR remains unknown. This lack of structural information prompted us to investigate the conformational properties of miniGαi1 in solution and compare them with those of full-length Gαi1.

### 2.2. GTP Binding and GTPase Activity of Gαi1 and miniGαi1

**Figure 2:**
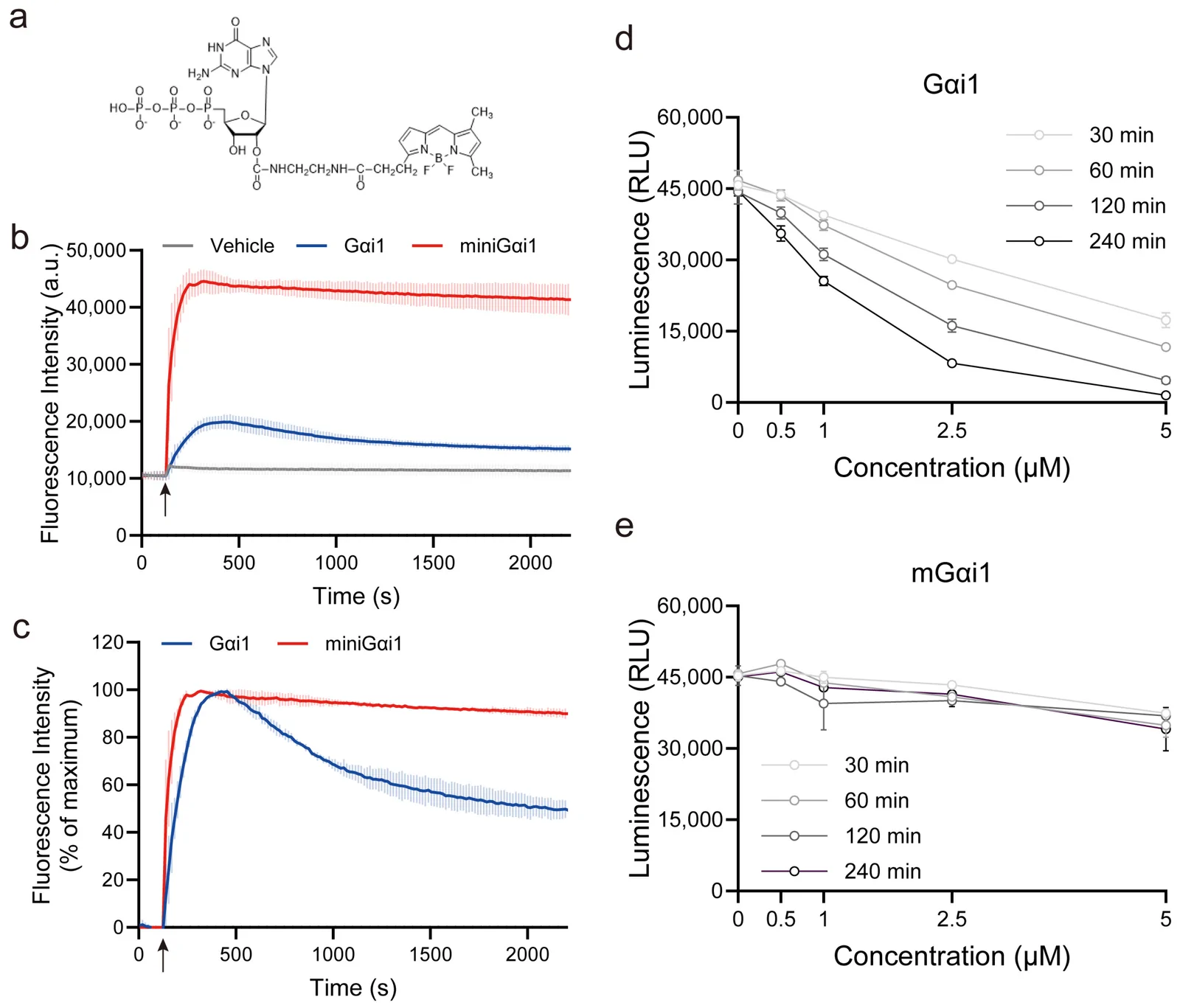
Comparison of GTP binding and GTPase activity between full-length Gαi1 and miniGαi1. (**a**) Chemical structure of BODIPY–FL–GTP. The BODIPY fluorophore is conjugated to the 2′ position of the ribose ring of GTP. (**b**) BODIPY–FL–GTP fluorescence assay. Full-length Gαi1 exhibited an increase in fluorescence followed by a gradual decrease, consistent with nucleotide binding and subsequent GTP hydrolysis. In contrast, miniGαi1 produced a larger fluorescence increase that remained elevated throughout the measurement period. The arrow indicates when the proteins were added. (**c**) Normalized fluorescence traces from the BODIPY–FL–GTP assay. Fluorescence intensities were normalized to the maximum signal to facilitate kinetic comparison. (**d**,**e**) GTPase activity of full-length Gαi1 (**d**) and miniGαi1 (**e**) measured using the GTPase-Glo™ assay. Luminescence was measured following incubation of full-length Gαi1 or miniGαi1 at the indicated protein concentrations for 30, 60, 120, or 240 min. Luminescence reflects the amount of residual GTP in the reaction and therefore inversely correlates with GTP hydrolysis. Data are presented as mean ± SD from 3 independent experiments.

To examine GTP binding and assess GTP hydrolysis activity, we performed a BODIPY–FL–GTP fluorescence assay (Figure 2a). In this system, the BODIPY–FL fluorophore is quenched when the nucleotide is free in solution, owing to the proximity between the fluorophore and the guanine ring facilitated by the flexible structure connecting them. Upon binding to Gα, the structure is fixed as an extended form, increasing the distance between the fluorophore and the guanine ring and resulting in enhanced fluorescence. However, the fluorescence intensity subsequently decreases following GTP hydrolysis due to the lower binding affinity of GDP compared with GTP, thereby enabling real-time monitoring of both GTP interaction and GTPase activity kinetics [10,11].

Addition of full-length Gαi1 to the BODIPY–FL–GTP solution resulted in an increase in fluorescence intensity followed by a gradual decrease (Figure 2b, blue line), consistent with binding of BODIPY–FL–GTP and subsequent hydrolysis of GTP to GDP. In contrast, addition of miniGαi1 induced a larger increase in fluorescence intensity, and the signal remained elevated throughout the measurement period (Figure 2b, red line).

To more precisely compare the kinetics, fluorescence intensities were normalized to the percentage of maximum fluorescence. miniGαi1 exhibited a faster fluorescence increase, with an estimated *k_obs_* of 0.0274 s^−1^ and a half-time of 25.3 s, compared with 0.0109 s^−1^ and 63.4 s, respectively, for full-length Gαi1. Thus, the apparent rate of fluorescence increase was approximately 2.5-fold higher for miniGαi1 than for full-length Gαi1, indicating faster interaction with BODIPY–FL–GTP. Furthermore, whereas the fluorescence signal of full-length Gαi1 gradually declined after reaching its maximum, the signal associated with miniGαi1 remained largely unchanged. These observations suggest enhanced nucleotide accessibility and reduced GTP hydrolysis activity in miniGαi1 relative to full-length Gαi1.

To further assess the GTP hydrolysis activity of full-length Gαi1 and miniGαi1, we employed the GTPase-Glo^TM^ assay [12]. This assay quantifies GTP remaining after the GTPase reaction through a coupled luminescence-based detection system. During the initial reaction, G proteins hydrolyze GTP to GDP, reducing the amount of residual GTP available for detection. Following the GTPase reaction, the remaining GTP is converted to ATP, which is subsequently used by luciferase to generate a luminescent signal. Thus, luminescence intensity is proportional to the amount of GTP remaining in the reaction and inversely reflects GTP hydrolysis activity. This assay enabled us to compare the GTP hydrolysis activities of full-length Gαi1 and miniGαi1 under the same experimental conditions. An important advantage of this approach is that it uses unmodified GTP rather than fluorescently labeled nucleotide analogs, avoiding potential effects of fluorophore conjugation on nucleotide recognition and hydrolysis. This assay therefore provides a complementary approach to fluorescent nucleotide-based assays for comparing the GTP hydrolysis activities of full-length Gαi1 and miniGαi1.

As expected, full-length Gαi1 showed a decrease in luminescence with increasing protein concentration and incubation time (Figure 2d), indicating GTP hydrolysis and a corresponding reduction in the amount of GTP remaining in the reaction. In contrast, miniGαi1 exhibited only a minimal decrease in luminescence (Figure 2e), suggesting substantially reduced GTPase activity. This result is consistent with the BODIPY FL–GTP fluorescence assay, which also suggested altered GTP hydrolysis by miniGαi1.

Taken together, these results suggest that miniGαi1 may exhibit enhanced GTP binding relative to full-length Gαi1, while its ability to hydrolyze bound GTP is substantially impaired.

### 2.3. Conformational Differences Between Gαi1 and miniGαi1

**Figure 3:**
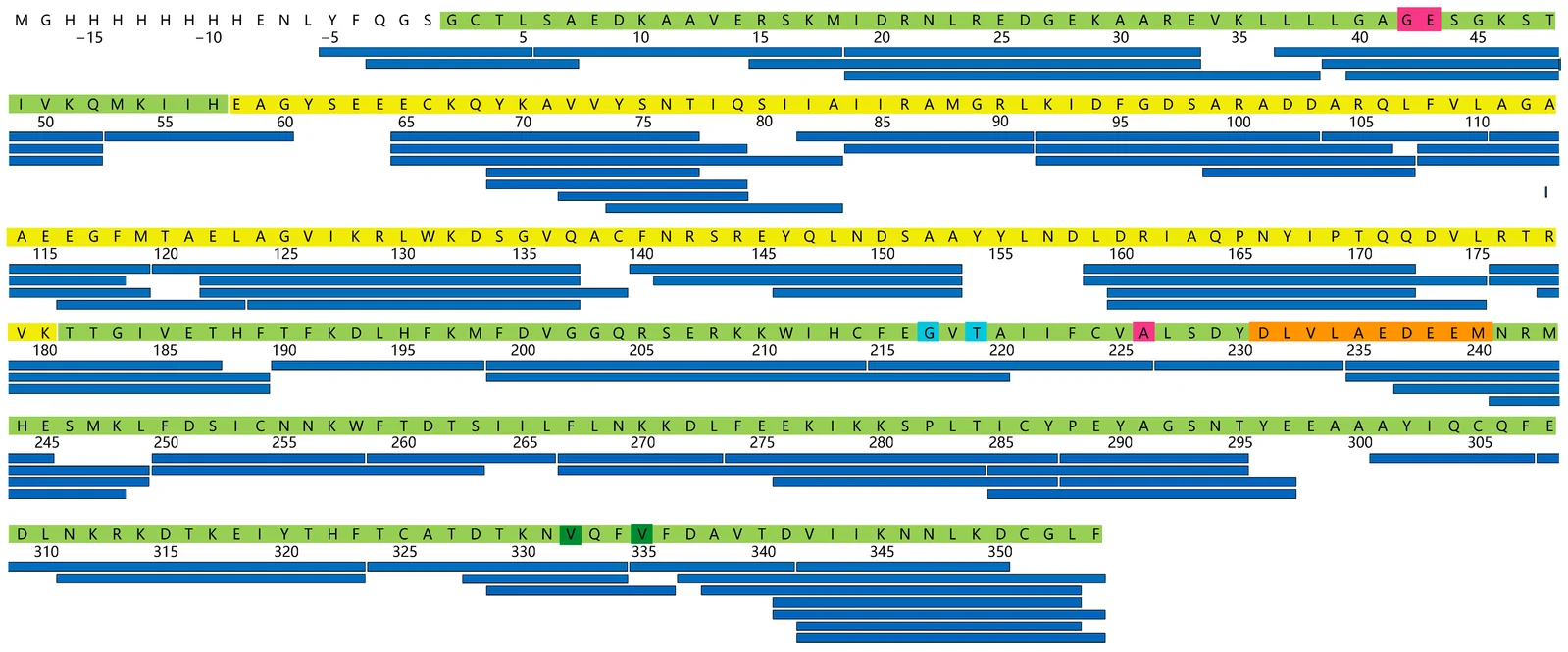
Peptic peptides used for HDX analysis. Selected peptic peptides used for HDX analyses are shown as blue sticks mapped onto the Gαi1 sequence. The Ras-like GTPase domain (RHD) and α-helical domain (AHD) are highlighted in pale green and yellow, respectively. Residues mutated in miniGαi1 are highlighted in colors corresponding to those used in Figure 1.

To understand the conformational differences between miniGαi1 and full-length Gαi1, we performed HDX-MS analysis. HDX-MS measures the exchange rate between hydrogen atoms within the peptide backbone amide and deuterium in the solvent and thus provides information on protein conformational dynamics and solvent accessibility [13]. We analyzed the HDX-MS profiles of miniGαi1 and GDP-bound Gαi1. The summary and complete HDX-MS dataset are provided in Dataset S1, and the peptic peptides used for the HDX-MS analysis are summarized in Figure 3.

**Figure 4:**
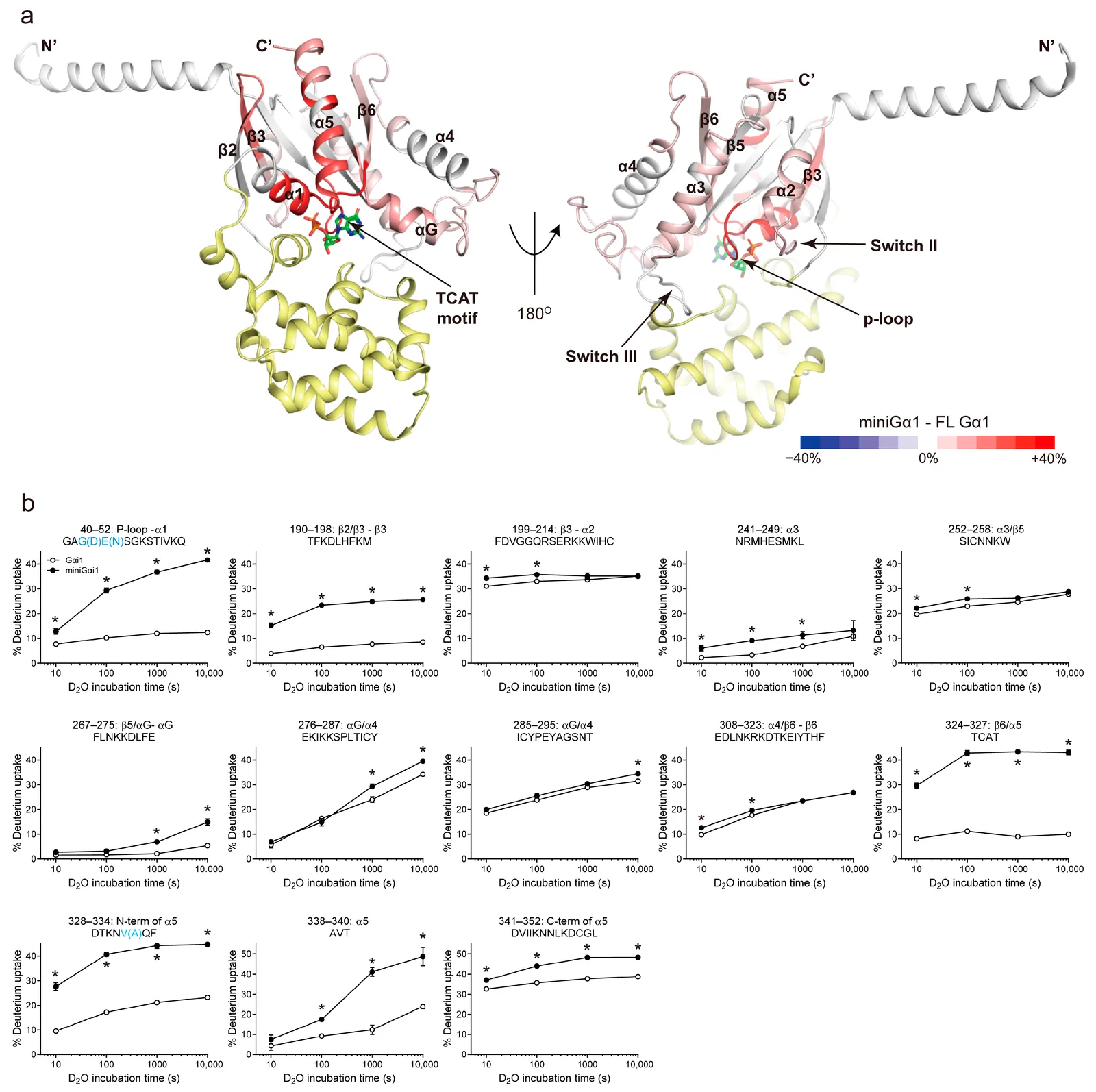
Conformational differences between full-length Gαi1 and miniGαi1 revealed by HDX-MS. (**a**) Differential HDX-MS was mapped onto the structure of GDP-bound Gαi1 (PDB 1GP2). Regions exhibiting differential deuterium uptake in miniGαi1 relative to full-length Gαi1 are colored according to the heat map scale shown in the color bar. The α-helical domain (AHD) is shown in yellow, and the nucleotide is shown as sticks. Color intensity represents the relative difference in deuterium uptake between miniGαi1 and full-length Gαi1. (**b**) Representative deuterium uptake plots for peptides showing significant differences between full-length Gαi1 (open circles) and miniGαi1 (filled circles). Residues mutated in miniGαi1 are highlighted in cyan within the peptide sequences. Data are presented as mean ± SD from 3 independent experiments. Asterisks indicate statistically significant differences between full-length Gαi1 and miniGαi1 at each time point (*p* < 0.05), as determined by an unpaired Student’s *t*-test.

Overall, miniGαi1 exhibited higher deuterium uptake than full-length Gαi1 across broad regions of the protein (Figure 4a). Notably, peptic peptides located near the nucleotide-binding pocket, including the P-loop through α1 (peptide 40–52), β3–α2 (switch II, peptide 199–214), α3 (peptide 241–249), β5/αG (peptide 267–275), β6/α5 (TCAT motif, peptide 324–327), and the N-terminal region of α5 (peptide 328–334), showed substantially higher deuterium uptake in miniGαi1 compared with full-length Gαi1 (Figure 4b). These observations suggest increased solvent accessibility and/or conformational flexibility around the nucleotide-binding pocket in miniGαi1, potentially arising from deletion of the AHD and reduced GDP interaction.

Although increased deuterium uptake can arise from either increased solvent accessibility or enhanced conformational flexibility, the time-dependent uptake profiles provide additional insight into the structural properties of individual regions (Figure 4b). For example, the P-loop–α1 region (peptide 40–52) of miniGαi1 exhibited a gradual increase in deuterium uptake over the D_2_O incubation period, suggesting that this region retains some degree of structural organization but exhibits greater solvent accessibility and/or conformational dynamics than in full-length Gαi1. In contrast, the β6–α5 region (peptide 324–327) and the N-terminal region of α5 (peptide 328–334) in miniGαi1 showed near-saturation of deuterium uptake (over 40%) within 100 s of D_2_O incubation. Given the approximately 40% back-exchange rate of our HDX-MS system, these results suggest rapid and extensive exchange in these regions, consistent with increased solvent exposure and/or conformational flexibility. Other regions near the nucleotide-binding pocket, including β3–α2 (switch II; peptide 199–214), α3 (peptide 241–249), and β5–αG (peptide 267–275), exhibited higher deuterium uptake in miniGαi1 than in full-length Gαi1 while retaining broadly similar time-dependent uptake profiles. This pattern suggests that these regions may preserve aspects of their overall structural organization while exhibiting increased solvent accessibility and/or conformational dynamics.

However, among these peptides, peptides 40–52 (P-loop) and 328–334 (the N-terminal region of α5) contain residues mutated in miniGαi1. Because amino acid substitutions can alter intrinsic hydrogen/deuterium exchange rates independent of protein conformation [14], the observed HDX differences in these peptides may not solely reflect structural dynamics. To evaluate this possibility, intrinsic exchange rates for all backbone amide hydrogens within these peptides were calculated using the HX software v3.0 developed by Englander and co-workers (http://hx2.med.upenn.edu/download.html, accessed on 21 July 2026) [14,15]. The predicted intrinsic exchange rates of peptide 40–52 were 1786.3 min^−1^ and 1877.0 min^−1^ for full-length Gαi1 and miniGαi1, respectively. Similarly, the predicted intrinsic exchange rates of peptide 328–334 were 711.3 min^−1^ and 757.3 min^−1^ for full-length Gαi1 and miniGαi1, respectively. Thus, the sequence-dependent differences in intrinsic exchange rates were relatively small (approximately 5–7%) compared with the experimentally observed HDX differences (approximately 20–30%). These results indicate that the elevated deuterium uptake observed in miniGαi1 cannot be fully explained by sequence-dependent effects and is therefore likely to reflect genuine differences in conformational dynamics.

Regions implicated in GPCR interaction, including the β2/β3 loop (peptide 190–198), α3/β5 (peptide 252–258), α4/β6 (peptide 308–323), and α5 (peptides 338–340 and 341–352), also exhibited higher deuterium uptake in miniGαi1 relative to full-length Gαi1 (Figure 4b). These results indicate that the receptor-binding regions, especially α5, are more conformationally dynamic in miniGαi1. In addition, higher deuterium uptake was observed in the αG/α4 region of miniGαi1 (Figure 4b; peptides 276–287 and 285–295) suggesting enhanced flexibility in this region as well.

## 3. Discussion

**Figure 5:**
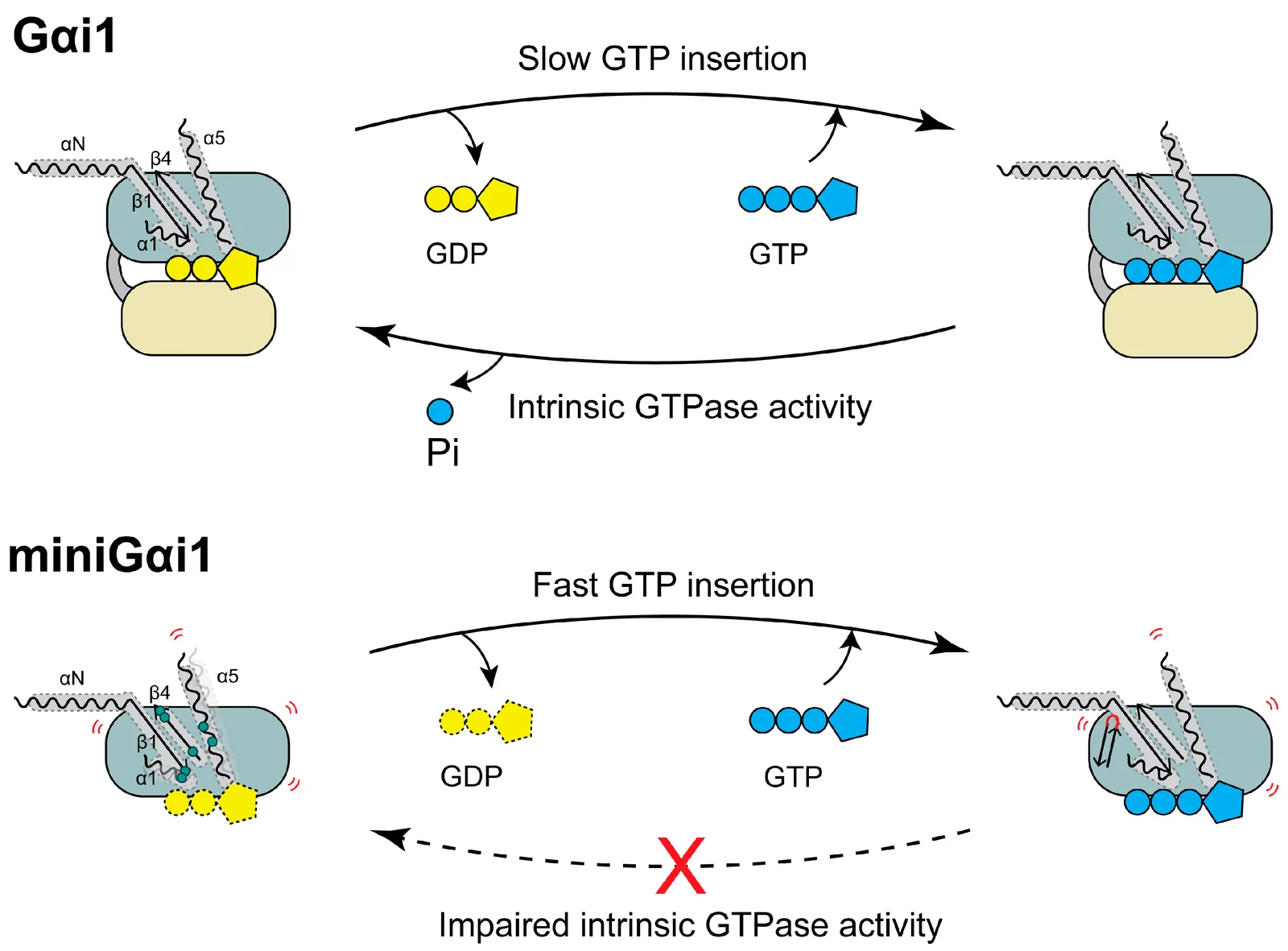
The functional differences between Gαi1 and miniGαi1. Schematic illustration comparing the proposed nucleotide exchange and GTP hydrolysis cycles of full-length Gαi1 (**upper panel**) and miniGαi1 (**lower panel**). Full-length Gαi1 contains an intact α-helical domain (AHD) and Ras-like domain (RHD), which together form the nucleotide-binding pocket and regulate GDP/GTP exchange and intrinsic GTPase activity. In contrast, deletion of the AHD and introduction of engineered mutations in miniGαi1 may alter structural constraints within the RHD, leading to increased conformational dynamics and nucleotide-pocket accessibility, as suggested by HDX-MS analysis. These structural alterations may contribute to faster apparent GTP binding but impaired intrinsic GTPase activity in miniGαi1. The αN, β1, α1, and α5 structural elements are shown in detail, with engineered mutations highlighted by pale green dots. Red curved lines indicate regions exhibiting enhanced conformational dynamics.

MiniG proteins have become indispensable tools for structural and pharmacological studies of GPCRs because of their enhanced stability and ability to stabilize active receptor conformations [6,7,8,9]. Despite their widespread use, the intrinsic structural and biochemical properties of miniG proteins have remained incompletely understood. Previous studies have investigated the effects of miniG proteins on the stability of GPCR–miniG complexes, their interactions with Gβγ, and their own structural stability [4,5]. However, their intrinsic nucleotide-binding properties, GTPase activity, and conformational dynamics have not been systematically characterized. In the present study, we compared miniGαi1 with full-length Gαi1 using complementary nucleotide assays and HDX-MS. Our results demonstrate that miniGαi1 exhibits distinct nucleotide interaction profiles and markedly increased conformational dynamics relative to full-length Gαi1 (Figure 5).

One of the most striking observations was the globally elevated deuterium uptake detected in miniGαi1. Increased HDX was observed not only in regions surrounding the nucleotide-binding pocket but also in multiple receptor-interacting regions. Because HDX reflects solvent accessibility and backbone dynamics, these results indicate that miniGαi1 exists in a more conformationally flexible state than GDP-bound full-length Gαi1. Importantly, enhanced HDX was detected in regions that do not contain engineered mutations, suggesting that the observed differences are not merely local effects of amino acid substitutions but represent global changes in protein dynamics.

The increased HDX observed around the nucleotide-binding pocket is consistent with the nucleotide-binding assays. The faster and more profound BODIPY-FL-GTP uptake to miniGαi1 than full-length Gαi1 along with higher HDX at the nucleotide-binding pocket suggests that miniGαi1 has relatively weak interaction with GDP and thus the purified miniGαi1 potentially exists in the empty state even when GDP is added. In contrast, once GTP is inserted, miniGαi1 stays as the GTP-bound form due to reduced GTPase activity. Therefore, when miniGαi1 is used for cellular assays, it is likely occupied with GTP because the cellular GTP concentration is higher than that of GDP [16].

A notable finding of this study is the increased conformational dynamics of α5 and other GPCR-interacting regions in miniGαi1. The α5 helix plays a central role in receptor recognition and undergoes substantial conformational rearrangement during GPCR-mediated activation [17,18,19,20]. The elevated HDX observed throughout α5 and neighboring receptor-contacting regions suggests that miniGαi1 adopts a broader conformational ensemble than GDP-bound full-length Gαi1. Such enhanced flexibility may facilitate productive coupling with active GPCRs and could contribute to the ability of miniG proteins to stabilize receptor active states in structural studies.

It is important to note that miniGαi1 differs from full-length Gαi1 not only by deletion of the AHD but also by the introduction of multiple stabilizing mutations and truncation of switch III. Therefore, the differences observed in this study cannot be attributed exclusively to AHD removal. Instead, they likely reflect the combined effects of domain deletion and protein engineering designed to optimize receptor coupling and biochemical stability. Future studies using intermediate constructs or molecular simulations may help dissect the individual contributions of these modifications.

Another important limitation is that no high-resolution structure of isolated miniGαi1 has been reported to date. Available structures have been determined exclusively in complex with GPCRs [7,9,21,22,23], making it difficult to infer the intrinsic conformational properties of miniG proteins in solution. The HDX-MS data presented here provide experimental evidence that miniGαi1 adopts a substantially different conformational landscape from GDP-bound full-length Gαi1, characterized by increased flexibility throughout the nucleotide-binding pocket and receptor-interacting interfaces.

Collectively, these findings suggest that the structural modifications incorporated into miniGαi1 reshape the conformational landscape of the RHD, leading to altered nucleotide interactions and enhanced conformational dynamics. Consequently, although miniG proteins faithfully mimic the receptor-bound state and remain invaluable for structural and pharmacological studies, caution should be exercised when interpreting experiments involving nucleotide binding, G protein activation, or conformational dynamics using these engineered constructs.

## 4. Materials and Methods

### 4.1. Materials

The protease inhibitor cocktail was purchased from Abcam (Cambridge, UK). Deuterium oxide was purchased from Cambridge Isotope Laboratories (Tewksbury, MA, USA). Chelating sepharose resin for Ni-IDA purification was purchased from Cytiva (Uppsala, Sweden). Sodium phosphate monobasic was purchased from Duchefa Biochemie B.V. (Haarlem, The Netherlands). HEPES, IPTG, leupeptin, lysozyme, ampicillin, and TCEP were purchased from GoldBio (St. Louis, MO, USA). Acetonitrile, H_2_O, and Methanol were purchased from J.T. Baker (Phillipsburg, NJ, USA). DNase I was purchased from Roche (Basel, Switzerland). BODIPY-FL-GTP was purchased from Thermo Fisher Scientific (Waltham, MA, USA). Acetic acid, benzamidine, formic acid, GDP, phosphoric acid, imidazole, and MgCl_2_ were purchased from Sigma–Aldrich (St. Louis, MO, USA).

### 4.2. Expression and Purification of Gαi1 and miniGαi1

Recombinant Gαi1 and miniGαi1 with an N-terminal 6×His tag were cloned into pET21a and transformed into *Escherichia coli* LOBSTR competent cells (Kerafast, Boston, MA, USA). Cells were grown in Terrific Broth containing ampicillin at 37 °C until the OD_600_ reached 0.6. Protein expression was induced with 0.03 mM IPTG for Gαi1 and 0.5 mM IPTG for miniGαi1, followed by incubation at 16 °C for 24 h. For protein purification, cell pellets were harvested and resuspended in lysis buffer (20 mM Tris-HCl pH 7.4, 300 mM NaCl, 2 mM MgCl_2_, 20 µM GDP, 1:1000 protease inhibitor cocktail, 2.5 µg/mL leupeptin, 10 µg/mL benzamidine, and 100 µM TCEP). Next, 3 mg/mL lysozyme was added to the mixture and incubated for 30 min at room temperature, followed by incubation with 10 μg/mL DNase I for another 30 min. The lysate was centrifuged at 18,000× *g* for 30 min at 4 °C, and the supernatant was supplemented with 20 mM imidazole, and then loaded onto a Ni-IDA column equilibrated with lysis buffer. Ni-IDA resin (Cytiva, Marlborough, MA, USA) was thoroughly washed with wash buffer (20 mM HEPES, pH 7.4, 100 mM NaCl, 2 mM MgCl_2_, 20 µM GDP, 1:1000 protease inhibitor cocktail, 2.5 µg/mL leupeptin, 10 µg/mL benzamidine, 100 µM TCEP, and 20 mM imidazole). Proteins were eluted with elution buffer (20 mM HEPES pH 7.4, 100 mM NaCl, 2 mM MgCl_2_, 20 µM GDP, 1:1000 protease inhibitor cocktail, 2.5 µg/mL leupeptin, 10 µg/mL benzamidine, 100 µM TCEP, and 200 mM imidazole). The eluate was further purified by size-exclusion chromatography using an ÄKTA FPLC system equipped with a Superdex-200 (10/300) column (Cytiva).

### 4.3. HDX-MS Analysis

For deuterium labeling, 4 μL of 50 μM Gαi1 or miniGαi1 was mixed with 26 µL of D_2_O buffer (20 mM HEPES pH 7.4, 100 mM NaCl, 2 mM MgCl2, 20 µM GDP, and 100 µM TCEP). The samples were incubated at room temperature for 10, 100, 1000, and 10,000 s and quenched with 30 µL of ice-cold quench buffer (60 mM NaH_2_PO_4_ pH 2.01, 20 mM TCEP, and 10% glycerol). The quenched samples were immediately snap-frozen on dry ice and stored at −80 °C. Non-deuterated samples were prepared by mixing 4 µL of protein samples with 26 µL of the corresponding H_2_O buffer (20 mM HEPES pH 7.4, 100 mM NaCl, 2 mM MgCl2, 20 µM GDP, and 100 µM TCEP), followed by quenching and snap-freezing as described above.

The quenched samples were thawed and immediately passed through an immobilized pepsin column (2.1 × 30 mm; Life Technologies, Carlsbad, CA, USA) for digestion at a flow rate of 100 µL/min in 0.05% formic acid in H_2_O at 12 °C. The peptic fragments were collected using a C18 VanGuard trap column (1.7 µm × 30 mm; Waters, Milford, MA, USA) for desalting with 0.05% formic acid in H_2_O and then separated using an Acuity UPLC C18 column (1.7 µm, 1.0 × 100 mm; Waters) at a flow rate of 40 µL/min with an acetonitrile gradient, with mobile phase B starting from 8% and increasing to 85% over 8.5 min. Mobile phase A was 0.15% formic acid in H_2_O and mobile phase B was 0.15% formic acid in acetonitrile. The pH of the buffers was adjusted to 2.5, and the system was maintained at 0.5 °C (except for pepsin digestion, which was performed at 12 °C) to minimize the back-exchange of deuterium. Mass spectrometry analyses were performed using a Xevo G2-XS QTof (Waters) as established previously [24,25]. ProteinLynx Global Server 2.4 (Waters) was used to identify peptides from the non-deuterated samples. DynamX 3.0 (Waters) was used to determine the level of deuterium uptake for each peptide by measuring the centroid of the isotopic distribution.

### 4.4. BODIPY-FL-GTP Assays

GTP binding/hydrolysis assays were performed using BODIPY-FL-GTP (Invitrogen, Waltham, MA, USA). The fluorophore was excited at 485 nm (bandwidth, 9 nm), and the emission spectrum was recorded at 510 nm (bandwidth, 20 nm) using a Synergy H1 Multimode Microplate Reader (BioTek, Winooski, VT, USA). The basal fluorescence of the imaging buffer (20 mM HEPES pH 7.4, 100 mM NaCl, 10 mM MgCl_2_, and 100 μM TCEP) was determined by measuring the fluorescence intensity without protein samples for 120 s. Ten μM Gαi1 or miniGαi1 was prepared in the same buffer (20 mM HEPES pH 7.4, 100 mM NaCl, 10 mM, 2 μM GDP, and 100 μM TCEP) and mixed with the imaging buffer with or without 1.2 μM BODIPY-FL-GTP. The change in fluorescence intensity was measured for 40 min at 10 s intervals in a 96-well black plate. All experiments were conducted at room temperature and repeated in 3 independent studies. The results were plotted using GraphPad Prism 8.0.

### 4.5. GTPAse Glo^TM^ Assay

The intrinsic GTPase activity of Gαi1 and miniGαi1 was analyzed using the GTPase-Glo^TM^ assay (Promega, Madison, WI, USA). G proteins prepared at various final concentrations (0, 0.5, 1, 2.5, 5 μM) were incubated with 5 μM GTP in assay buffer (20 mM HEPES pH 7.5, 100 mM NaCl, 10 mM MgCl_2_, 1 mM DTT, and 1 μM GDP) for 30, 60, 120 and 240 min. After reaction, GTPase-Glo reagent supplemented with 5 μM ADP was added to the sample, and the mixtures were incubated for an additional 30 min at room temperature to convert the remaining GTP into ATP. Subsequently, the detection reagent (Promega) was added and incubated for 10 min at room temperature. Luminescence was measured using a Synergy H1 Multimode Microplate Reader (BioTek, Winooski, VT, USA). All experiments were performed in three independent experiments, and the data were analyzed using GraphPad Prism 8.0.

### 4.6. Statistical Analysis

Data from the BODIPY FL–GTP assay, GTPase-Glo™ assay, and HDX-MS analyses are presented as mean ± SD from three independent experiments. To characterize the apparent kinetics of nucleotide interactions in the BODIPY FL–GTP assay, the initial increase in fluorescence was fitted to a single-exponential association model to estimate the observed rate constant (*k_obs_*) and the corresponding half-time (*t*_1/2_). Differences in HDX-MS data between full-length Gαi1 and miniGαi1 were evaluated using an unpaired Student’s *t*-test. A *p* value < 0.05 was considered statistically significant.

### 4.7. Use of Generative Artificial Intelligence (GenAI)

GenAI (ChatGPT-5.6 Terra) was used for English editing.

## Data Availability

The data presented in this study are available on request from the corresponding author.

## Supplementary Materials

The following supporting information can be downloaded at https://www.mdpi.com/article/10.3390/ijms27219819/s1.

## Abbreviations

The following abbreviations are used in this manuscript:

GDP	Guanosine diphosphate
GTP	Guanosine triphosphate
GTPγS	Guanosine-5′-(γ-thio)-triphosphate
HDX-MS	Hydrogen–deuterium exchange mass spectrometry
GPCR	G protein-coupled receptor
